# Multicenter Study of Creatinine- and/or Cystatin C-Based Equations for Estimation of Glomerular Filtration Rates in Chinese Patients with Chronic Kidney Disease

**DOI:** 10.1371/journal.pone.0057240

**Published:** 2013-03-19

**Authors:** Jia-fu Feng, Ling Qiu, Lin Zhang, Xue-mei Li, Yu-wei Yang, Ping Zeng, Xiu-zhi Guo, Yan Qin, Hong-chun Liu, Xing-min Han, Yan-peng Li, Wei Xu, Shu-yan Sun, Li-qiang Wang, Hui Quan, Li-jun Xia, Hong-zhang Hu, Fang-cai Zhong, Rong Duan

**Affiliations:** 1 Laboratory Medicine, Mianyang Central Hospital, Mianyang, Sichuan Province, China; 2 Kidney Internal Medical Department, Mianyang Central Hospital, Mianyang, Sichuan Province, China; 3 Laboratory Medicine, Peking Union Medical College Hospital, Bejing, China; 4 Kidney Internal Medical Department, Peking Union Medical College Hospital, Bejing, China; 5 Laboratory Medicine, The First Affiliated Hospital of Zhengzhou University, Zhengzhou, Henan Province, China; 6 Department of Nuclear Medicine, The First Affiliated Hospital of Zhengzhou University, Zhengzhou, Henan Province, China; 7 Laboratory Medicine, The First Bethune Hospital of Jilin University, Jilin, Jilin Province, China; 8 Laboratory Department, Nuclear Industrial 416 Hospital, Chengdu, Sichuan Province, China; 9 Laboratory Department, The First People's Hospital of Neijiang, Neijiang, Sichuan Province, China; 10 Kidney Internal Medical Department, The First People's Hospital of Neijiang, Neijiang, Sichuan Province, China; Gentofte University Hospital, Denmark

## Abstract

**Objective:**

To establish equations for the estimation of glomerular filtration rates (eGFRs) based on serum creatinine (SCr) and/or serum cystatin C (SCysC) in Chinese patients with chronic kidney disease (CKD), and to compare the new equations with both the reference GFR (rGFR) and the literature equations to evaluate their applicability.

**Methods:**

The 788 Chinese CKD patients were randomly divided into two groups, the training group and the testing group, to establish new eGFR-formulas based on serum CysC and to validate the established formulas, respectively. ^99m^Tc-DTPA clearance (as the rGFR), serum Cr, and serum CysC were determined for all patients, and GFR was calculated using the Cockcroft-Gault equation (eGFR1), the MDRD formula (eGFR2), the CKD-EPI formulas (eGFR3, eGFR4), and the Chinese eGFR Investigation Collaboration formulas (eGFR5, eGFR6). The accuracy of each eGFR was compared with the rGFR.

**Results:**

The training and testing groups' mean GFRs were 50.84±31.36 mL/min/1.73 m^2^ and 54.16±29.45 mL/min/1.73 m^2^, respectively. The two newly developed eGFR formulas were fitted using iterative computation: 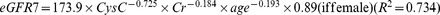 and 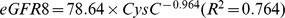. Significant correlation was observed between each eGFR and the rGFR. However, proportional errors and constant errors were observed between rGFR and eGFR1, eGFR2, eGFR4, eGFR5 or eGFR6, and constant errors were observed between eGFR3 and rGFR, as revealed by the Passing & Bablok plot analysis. The Bland-Altman analysis illustrated that the 95% limits of agreement of all equations exceeded the previously accepted limits of <60 mL/min •1.73 m^2^, except the equations of eGFR7 and eGFR8.

**Conclusion:**

The newly developed formulas, eGFR7 and eGFR8, provide precise and accurate GFR estimation using serum CysC detection alone or in combination with serum Cr detection. Differences in detection methods should be carefully considered when choosing literature eGFR equations to avoid misdiagnosis and mistreatment.

## Introduction

Chronic kidney disease (CKD) is a serious public health problem worldwide and is usually defined as kidney damage or decreased kidney function with glomerular filtration rates (GFRs) of less than 60 mL/min per 1.73 m^2^ for 3 months or longer, regardless of cause [1]–[3]. Based on GFR, CKD is classified into different stages that require stage-specific management. Therefore, accurate measurement of GFR is critical to evaluate the patient's renal function. Currently, the “gold standard” for GFR determination is to measure the clearance of exogenous substances, such as inulin, iohexol, ^51^Cr-EDTA, ^99m^Tc-DTPA and ^125^I-iothalamate [4]. However, these measurements are not only time-consuming, labor-intensive and expensive but also require the administration of rare substances; thus, these methods are not routinely used [5]. Therefore, serum or plasma creatinine levels have become the most commonly used markers for GFR determination because of the simplicity and lower costs of this method [6], [7]. GFR can be calculated based on plasma or serum creatinine using the Cockcroft-Gault or the Modification of Diet in Renal Disease (MDRD) study equations [7], [8]. However, using plasma or serum creatinine has significant disadvantages, such as the inability to measure renal function correctly when impairment is 50% or less [7]. Creatinine generation is proportional to muscle mass and related to an individual's age, sex, race and weight [4], [7]. As a result, an increase in serum creatinine may not be observed until a substantial decrease in GFR has occurred.

Cystatin C (CysC) is a cysteine protease inhibitor with a molecular mass of 13 kDa [9]. It has been shown that cystatin C is a more sensitive marker of GFR changes than serum creatinine [10] because its levels are not affected by muscle mass, age, inflammation, fever or exogenous agents [11]. CysC is produced at a constant rate and cleared solely by glomerular filtration [11], and it can be measured easily with particle-enhanced nephelometric immunoassay (PENIA) [12] or particle-enhanced turbidimetric immunoassay (PETIA) [13]. Large amount of studies have shown that CysC is superior to SCr in predicting the function of kidney [14]–[18]. Therefore, CysC has been used as an alternative endogenous serum marker of GFR [19], and many formulas for GFR estimation have been developed based upon serum CysC determination [20], [21]. However, these formulas were all established based on a small sample and outside of a laboratory, and the “gold standard” measurement of GFR and CysC is also very inconsistent. In addition, these equations have been established in Western populations and they may or may not be suitable for the Chinese population, which requires clinical validation.

The PETIA-CysC method is becoming more common in clinical practice because of its lower cost and more rapid detection than the PENIA-CysC method [22]–[25]. For creatinine measurement, the enzymatic method (enzymatic-Cr) is widely used in the clinical laboratory because of its low chance of cross-contamination and stable results [26]–[28]. In the present study, we applied enzymatic-Cr and PETIA-CysC to determine the creatinine levels and CysC levels, respectively. Following the measurements, we established the eGFR equations based on the levels of CysC and/or serum Cr(SCr) in Chinese CKD patients with multi-center cooperation and evaluated the applicability of these GFR estimating equations.

## Materials and Methods

### Subjects

A total of 788 CKD patients who were referred by nephrologists, diabetologists, cardiologists or general internists were selected from 6 general hospitals between October 2010 and December 2011 in different areas of China, including 421 males (aged 50.4±15.7 years) and 367 females (age,d 51.6±16.6 years). These were 355 patients from North China(Jilin and Beijing, China), 82 patients from Central China(Henan,China), and 351 patients from South China(Mianyang, Chendu and Neijiang, Sichuan, China).All patients met the diagnostic criteria of NKF-KDOQI CKD [1], and the following patients were excluded during the selection: (1) patients with acute kidney disease or acute renal insufficiency; (2) dialysis patients; (3) patients with merger edema, pleural effusion, ascites, thyroid disease, or viral hepatitis (carriers were exceptional); (4) malnourished patients (lower than normal protein, blood urea or urine conductivity); (5) disabled patients; and (6) patients using antibacterial drugs, especially trimethoprim and cimetidine.

A total of 687 cases were chosen randomly for equation development (training group), which included 358 males (aged 50.2±15.6 years) and 329 females (aged 51.5±16.7 years). The remaining 101 cases were assigned to the equation validation (testing group), including 63 males (aged 51.7±16.1 years) and 38 females (aged 52.0±16.1 years).

The study was approved by the Medical Ethics Committee of Mianyang Central Hospital, Peking Union Medical College Hospital, The First Affiliated Hospital of Zhengzhou University, The First Bethune Hospital of Jilin University, Nuclear Industrial 416 Hospital, or The First people's Hospital of Neijiang, and written informed consent was obtained from all subjects.

### Sample collection

Blood samples were collected at 8:00 AM. Approximately 5 ml of blood was collected into a BD Vacutainer® SST^TM^ II ADVANCE tube (Becton Dickinson, USA) for analysis of CysC and SCr concentrations. After one hour, blood samples were centrifuged at 3000 rpm for 15 min, and serum samples were collected and stored at −80°C until analysis within 480 hours.

### Detection methods

Because of the distribution of subjects in different regions, reference GFR (rGFR) values can only be determined independently in each study institute. In order to make the inter-institutes variance as small as possible, a identical research program among study institutes was established, which including researcher training, ^99m^Tc-DTPA drug selection (radiochemical purity greater than 95%, percentage of ^99m^Tc-DTPA bound to plasma protein less than 5%), patients' preparation, intravenous injection, blood sampling time point and procedure, regular maintenance of instrument, and radioactivity measurement.

Reference GFR (rGFR) for each subject was measured when blood samples were collected. In all patients included in the six participating study institutes,^99m^Tc-DTPA clearance was measured as a rGFR. rGFR was measured by the dual plasma sampling method [29], standardized by body surface area (BSA), and resulted in the rGFR: rGFR (mL/min per 1.73 m^2^) = [Dln (P1/P2)/(T2-T1)] exp {[(T1lnP2)−(T2lnP1)]/(T2−T1)}×0.93×1.73/BSA, where D is dosage of drug injected. P1 and P2 is plasma activity at T1(first blood sampling) and T2(second blood sampling,), respectively. Units for D, P1 and P2 were cpm/ml, for T1, T2 was minute.Unlike rGFR, SCr and CysC levels were measured in a single laboratory, using a 7600–020 Automatic Analyzer (Hitachi, Japan), CysC concentrations was measured by PETIA-CysC method and SCr concentrations were measured by an enzymatic method according to the determination of glycine after enzymatic conversion of creatinine to glycine that can be traced back to IDMS, which kits produced by Sichuan Maker Biotechnology Co., Ltd.(Sichuan, China), but CysC reagents are original equipment manufacture(OEM) products that were obtained from Gentian (Moss, Norway), its calibration can be traced back to ERM-DA471/IFCC. Serum CysC measurements were performed using the following instrument settings: Primary wavelength: 546 nm; Secondary wavelength: 700 nm. Temperature: 37°C; Read points: 19–34; sample blank position 16 and spline calibration method. 157 µL assay buffer (reagent 1), and 3.5 µL sample were mixed with 52 µL anti-cystatin C immunoparticles (reagent 2),. The sensitivity of the assay for CysC was 0.03 mg/L. The intra-assay CV was 2.25% (mean, 0.92 mg/L; n = 20), and the day-to-day CV was 3.18% (mean, 0.68 mg/L; n = 30). SCr measurements were performed using the following instrument settings: Primary wavelength: 546 nm; Secondary wavelength: 700 nm. Temperature: 37°C; Read points: 17–34; sample blank position 16 and linear calibration method. 155 µL enzyme working solution (reagent 1), and 2.0 µL sample were mixed with 26 µL chromogen solution (reagent 2),. The sensitivity of the assay for SCr was 2.40 µmol/L. The intra-assay CV was 1.12% (mean, 135.6 µmol/L; n = 20), and the day-to-day CV was 1.33% (mean, 382.5 µmol/L; n = 30).

### eGFR calculation formula

**Cockcroft-Gault formula [30]:**

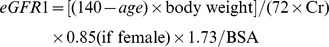


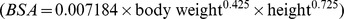



**Simplified MDRD formula [31]:**

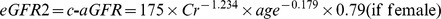



**MDRD/CKD-EPI formula [32]:**

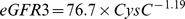


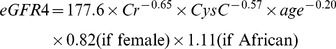



**eGFR formula of Chinese collaborative group [33]:**

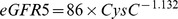


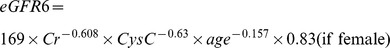



Unified measurement units were used in the above 6 formulas and the following eGFR7 and eGFR8 formulas to facilitate comparative analyses: Cr: mg/dl; CysC: mg/L; BSA: m^2^; age: years; body weight: kg; height: cm.

### Statistical analyses

The measurement data are presented as the means±SD, median and range. The differences between genders were estimated by the student t-test for the normal distribution data or the Kolmogorov-Smirnov test for non-normally distribution data.The GFR estimation equations were established based on the Spearman correlation analysis and non-linear regression of parameters including rGFR, CysC, Cr and age. The differences between GFR estimates and rGFR were tested by ANOVA. The consistency was tested by correlation analysis and Bland-Altman analysis, and the previously accepted tolerances was defined as 60 mL/min •1.73 m^2^ [34]. The differences between rGFR and eGFR were analyzed by Passing-Bablok regression analysis. The difference distributions between eGFR and rGFR were performed by Mountain plot. The deviations are shown as the area between the Bland-Altman regression line of difference and the zero difference line. The precision is represented by the coefficient of repeatability (CR). The accuracies are shown as P_15_, P_30_, or P_50_, which represented the proportion of eGFR within 15%, 30%, and 50% of rGFR (±15%, ±30%, or ±50%). Kappa statistics were used to evaluate the agreement between stages classification from rGFR method and other eGFR methods. The K value can be interpreted as follows: poor agreement (<0.20), fair agreement (0.21–0.40), moderate agreement (0.41–0.60), good agreement (0.61–0.80) and very good agreement (0.81–1.0).The PASW Statistics 18.0 (SPSS Inc., Somers, NY, USA) and MedCalc11.5 (MedCalc Software, Mariakerke, Belguim) software products were used for these statistical analyses. Differences with *P*<0.05 are considered statistically significant.

## Results

### eGFR curve fitting based on the concentrations of serum CysC and serum Cr

The basic characteristics of the 788 patients are listed in Table 1. The datasets from the equation development group were tested by Spearman correlation analysis, which revealed negative correlations of rGFR with age, CysC and Cr (r = −0.212, −0.855 and −0.809, all *P* = 0.000). Further non-linear regression fitting was performed for rGFR with age, CysC and Cr, and iterative calculations were used to establish the equation:
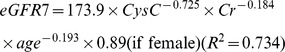



**Table 1:** Demographic characteristics of the 788 Chinese CKD patients (up line: mean±SD, down line: median, range).

	Total	Male	Female	*t*/*z*	*P*
Training group (n = 687)					
n	687	358	329	-	-
Age (year)	50.8±16.1 51.0, 19.0–87.0	50.2±15.6 50.0, 19.0–87.0	51.5±16.7 52.0, 19.0–87.0	−1.056	0.293
Height (cm)	164.4±7.7 165.0, 148.0–184.0	168.8±6.6 170.0, 150.5–184.0	159.7±5.7 160.5, 148.0–175.0	19.514	0.000
Weight (kg)	63.5±11.7 63.0, 32.5–110.0	68.5±11.5 68.0, 46.0–110.5	59.2±9.4 57.0, 32.5–99.0	12.912	0.000
BSA (m^2^)	1.69±0.17 1.70, 1.18–2.28	1.78±0.16 1.79, 1.41–2.28	1.60±0.13 1.59, 1.18–2.05	16.470	0.000
rGFR(mL/min·1.73 m^2^)	50.84±31.36 44.19, 3.51,166.00	50.38±30.26 45.23, 3.51,147.91	51.33±32.56 43.50, 3.95,166.00	0.597	0.868
CysC (mg/L)	2.31±1.44 1.88, 0.59–8.62	2.39±1.50 1.95, 0.59–8.62	2.22±1.37 1.80, 0.60–7.44	−1.056	0.215
Cr(mg/dl)	2.78±2.78 1.73, 0.40–19.77	2.94±2.79 1.84, 0.46–19.77	2.60±2.78 1.69, 0.40–15.06	−1.585	0.113
Testing group (n = 101)					
n	101	63	38	–	–
Age (year)	51.8±16.0 51.0, 22.0–86.0	51.7±16.1 49.0, 22.0,86.0	52.0±16.1 56.5, 25.0,84.0	−0.101	0.920
Height (cm)	165.9±8.7 165.5, 150.0–182.0	170.0±7.5 170.0, 153.5–182.0	159.1±5.6 159.5, 150.0–174.0	8.331	0.000
Weight (kg)	65.6±14.0 63.0, 41.5–108.0	70.9±14.0 72.5, 47.0–108.0	56.8±8.9 55.0, 41.5–86.5	6.184	0.000
BSA (m^2^)	1.72±0.21 1.72, 1.34–2.27	1.81±0.19 1.82, 1.47–2.27	1.58±0.12 1.56, 1.34–1.88	7.517	0.000
rGFR(mL/min·1.73 m^2^)	54.16±29.45 47.85, 10.49–148.12	55.02±30.93 48.51, 10.49–148.12	52.73±27.16 44.52, 15.34–139.43	−0.284	0.776
CysC (mg/L)	2.13±1.41 1.79, 0.66–7.22	2.30±1.55 1.85, 0.66–7.22	1.85±1.09 1.74, 0.76–6.39	−0.964	0.335
Cr (mg/dl)	2.39±2.86 1.56, 0.48–23.34	2.46±2.25 1.63, 0.52–20.46	2.28±3.69 1.30, 0.48–23.34	−1.157	0.247

Note: training group *vs.* testing group; all measured indicators had *P*>0.05 (*t* represents t values of the student *t*-test. *z represents z values of Kolmogorov-Smirnov test.)

In addition, the non-linear regression equation of rGFR was established using the CysC single index:
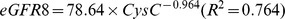



### Analysis of the differences between the rGFR and eGFR values estimated by the equations

**Figure 1:**
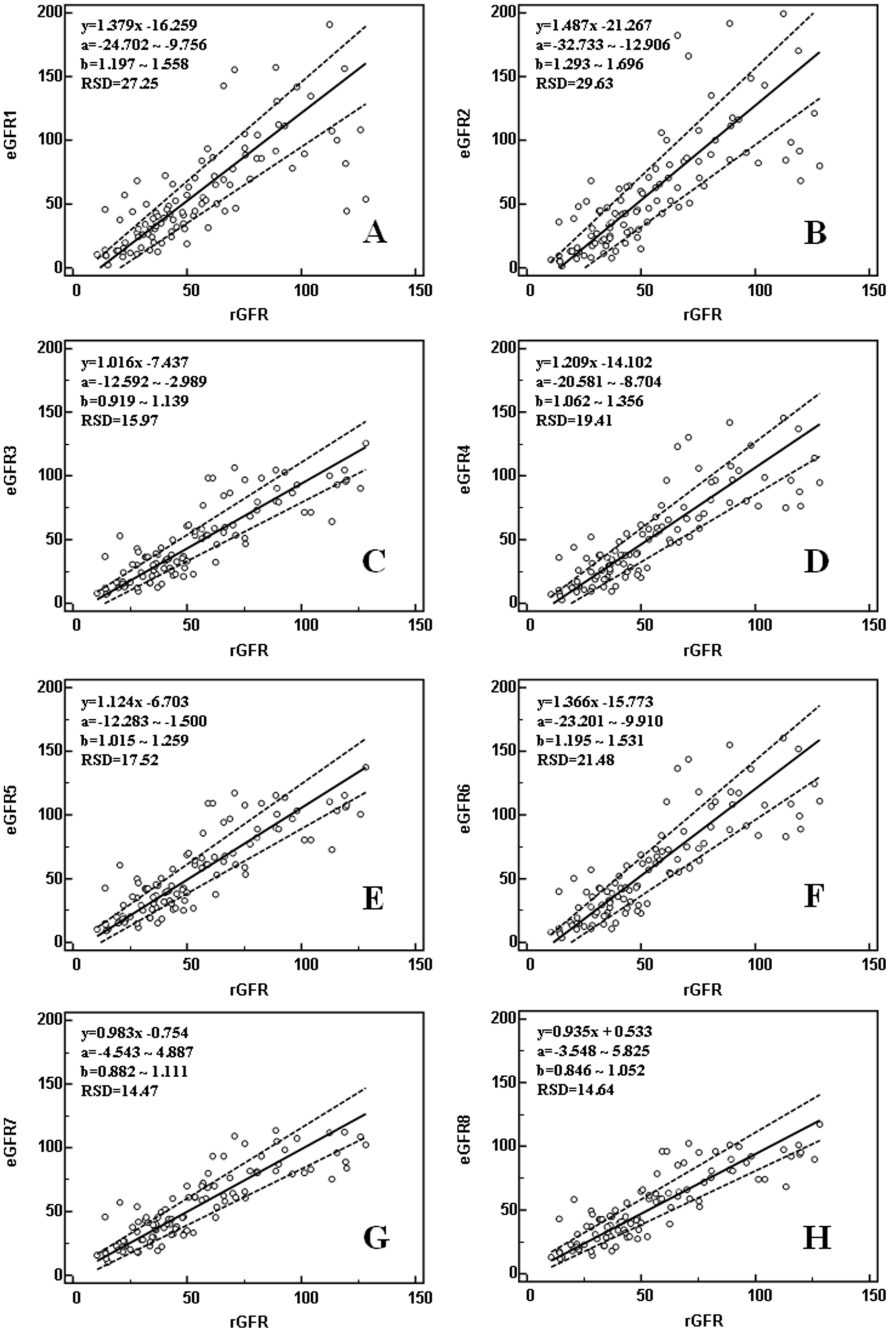
Passing-Bablok plot to analyze and compare eGFR with rGFR. (a: 95% confidence interval for the intercept; b: 95% confidence interval for the slope; RSD: residual standard deviation; Cusum test, all *P*>0.05)

The aforementioned 8 equations were used to calculate the eGFRs of the 101 cases in the testing group, which were compared to the rGFR using ANOVA (Table 2). The calculated eGFR values were highly correlated (*P* = 0.000) with, but not significantly different (F = 0.812, *P* = 0.592) from, the rGFR values. Further Passing & Bablok analysis revealed that each eGFR value had no apparent linear deviation from the rGFR values (all *P*>0.05). However, among the eGFR values, eGFR1, eGFR2, eGFR4 and eGFR5 all showed significant proportional differences (the 95% CI of slopes did not include B = 1) and significant constant differences (the 95% CI of intercepts did not include A = 0) with eGFR6 (Fig 1-A, B, D-F); eGFR2 showed the most significant errors of both types (a = −21.267, b = 1.487), and eGFR3 also showed a highly significant constant error (Fig 1-C). Only two new equations of the eight did not show significant differences in the proportional errors and the constant errors.

**Table 2:** Overall limits of agreement between eGFR and rGFR (n = 101).

	mean±SD	correlation analysis		Bland-Altman analysis			
		*r*	*P*	Mean differences	95% AL	acceptable limits*	Out of limitsn (%)
rGFR	54.16±29.45	-	-	-	-	-	-
eGFR1	56.60±29.70	0.7734	0.0000	−2.4	−51.8–46.9	98.7	6(5.94)
eGFR2	58.21±44.50	0.7774	0.0000	−4.1	−59.8–51.7	111.5	4(3.96)
eGFR3	48.44±±30.04	0.8600	0.0000	5.7	−25.2–36.6	61.8	5(4.95)
eGFR4	51.00±35.10	0.8510	0.0000	3.2	−33.0–39.3	72.3	6(5.94)
eGFR5	55.06±32.68	0.8600	0.0000	−0.9	−33.7–31.9	65.6	5(4.95)
eGFR6	56.96±39.07	0.8556	0.0000	−2.8	−43.2–37.6	80.8	5(4.95)
eGFR7	54.47±28.06	0.8729	0.0000	−0.3	−28.8–28.2	57.0	7(6.93)
eGFR8	52.59±27.07	0.8591	0.0000	1.6	−28.2–31.3	59.5	7(6.93)

Note: Units are mL/min·1.73 m^2^; 95% AL, 95% agreement limits. *Acceptable tolerance for the difference between rGFR and eGFR was defined as 60 mL/min/1.73 m^2^. *r* is the Person's correlation coefficient between eGFR and rGFR.

### Consistency analysis of the estimated eGFR values and the rGFR values

**Figure 2:**
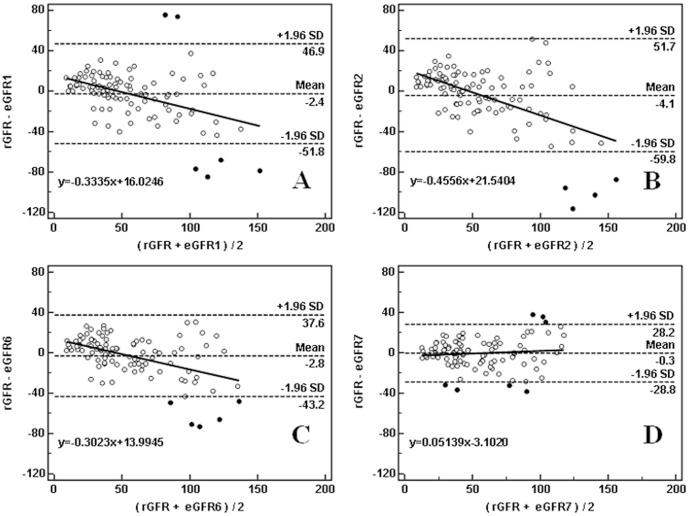
Altman-Bland plot: comparison between eGFR and rGFR (n = 101).

As shown in Table 3, when the eGFR values were compared with the rGFR values, the most significant deviation was observed for eGFR2 (Figure 2-B, 3036 arbitrary units), followed by eGFR1 (Fig. 2-A, 2045 arbitrary units) and eGFR6 (Figure 2-C, 1435 arbitrary units). The deviations of the other 5 eGFR equations were similar to each other, and eGFR7 showed the smallest deviation (Figure 2D, 367 arbitrary units).

**Table 3:** Bias, precision and accuracy of eGFR compared with rGFR (n = 101).

	Bias*	CR** mL/min·1.73 m^2^	Accuracy (%)		
			P_15_	P_30_	P_50_
eGFR1	2045	49.3	36.63	55.45^a,d^	77.23^a,c^
eGFR2	3036	55.8	23.76^a,c^	43.56^a,c^	73.27^a,c^
eGFR3	628	30.9	34.65	63.37	86.14^b^
eGFR4	681	36.1	36.63	63.37	81.19^a,d^
eGFR5	490	32.8	39.60	65.35	89.11
eGFR6	1435	40.4	31.68	59.41^b^	82.18^a,d^
eGFR7	367	28.5	41.58	74.26	95.05
eGFR8	377	29.7	41.58	72.28	93.07
*χ* ^2^	–	–	10.812	28.341	31.399
*P*	–	–	0.147	0.000	0.000

* Bias, the area between the Bland-Altman regression line and the zero difference line; arbitrary unit, i.e., (mL/min·1.73 m^2^)^2^. **CR, Coefficient of Repeatability, equal to the difference between the mean difference and the 95% upper limit of agreement. a: *vs.* eGFR7, by Pearson *χ* ^2^ test, *P*<0.01: b: *vs*. eGFR7, by Pearson *χ* ^2^ test, *P*<0.05; c: *vs.* eGFR8, by Pearson *χ* ^2^ test, *P*<0.01; d: *vs.* eGFR8, by Pearson *χ* ^2^ test, *P*<0.05.

The performed accuracy showed significant differences in P_30_ and P_50_ among various eGFR equations (χ^2^ = 28.341 and 31.399, respectively; *P* = 0.000 for both). The highest performed accuracies in P_30_ and P_50_, 74.26% and 95.05%, respectively, were observed for eGFR7, followed by eGFR8, with 72.28% and 93.07%, respectively. eGFR2 showed the lowest performed accuracies at 43.56% and 73.27%, respectively, followed by eGFR1 at 55.45% and 77.23%, respectively.

Overall, eGFR7 showed the highest performed accuracy for rGFR estimation (Figure 2-B, CR = 28.5 mL/min • 1.73 m^2^).

As shown in Table 2, the Bland-Altman analysis revealed that the percentage of each eGFR value falling outside the consistency limit was between 3.96 and 6.93%, which were not significantly different (χ^2^ = 1.483, *P* = 0.983). However, the eGFR3, eGFR4 and eGFR8 values were slightly lower than the rGFR values (positive deviation), while others were higher. The consistency limits of six equations were all higher than the previously accepted tolerances (<60 mL/min • 1.73 m^2^), while those of eGFR7 and eGFR8 were within the previously accepted tolerances.

**Figure 3:**
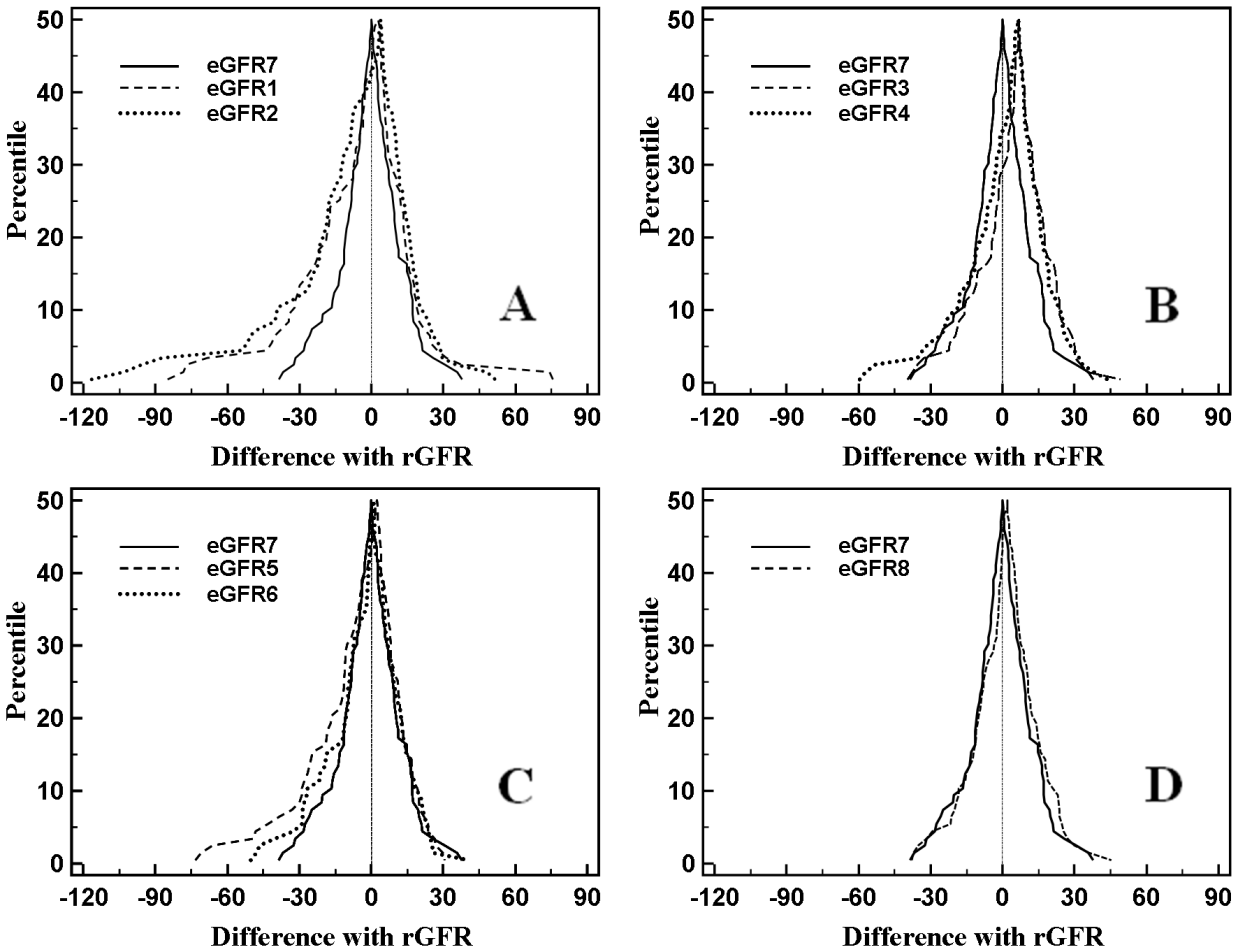
Mountain plot: comparison between newly developed eGFR formulas and various literature eGFR formulas.

Using the Mountain chart, the newly developed formulas, eGFR7 and eGFR8, and 6 previously reported formulas in the literature were used to estimate the differences in the consistency between the estimated GFR values and the rGFR values (Figure 3). The median deviation of the distribution curve (M, i.e., P_50_) was used to show their central tendency, with the range of *P*_5_–*P*_95_ (R*_P_*_5–*P*95_) representing the degree of dispersion (Table 4).

**Table 4:** Percentiles (*P*) of difference between eGFR and rGFR (mL/min • 1.73 m^2^).

	*P*_5_	*P*_10_	*P*_25_	*P*_50_(*M*)	*P*_75_	*P*_90_	*P*_95_
eGFR1	−42.52	−32.93	−14.24	2.85	12.12	19.06	25.91
eGFR2	−52.86	−39.34	−17.52	3.77	13.36	21.20	28.15
eGFR3	−22.21	−15.85	−2.06	6.90	14.67	23.74	29.88
eGFR4	−31.91	−19.84	−5.28	6.63	14.80	23.82	28.48
eGFR5	−30.58	−26.63	−9.25	0.88	9.42	18.96	23.46
eGFR6	−45.36	−29.05	−11.39	2.51	11.19	19.44	23.73
eGFR7	−27.94	−17.96	−8.31	0.01	8.89	17.13	21.00
eGFR8	−25.04	−18.27	−7.89	2.09	10.58	21.73	24.90

Compared with eGFR7 (*M* = 0.01, R*_P_*_5–*P*95_ = 48.94 mL/min • 1.73 m^2^), eGFR1-6 all showed a larger M of deviation with rGFR (the *M* were 2.85, 3.77, 6.90, 6.63, 0.88 and 2.51 mL/min • 1.73 m^2^, respectively) and wider curve distribution (the R*_P_*_5-*P*95_ were 68.43, 81.01, 52.09, 60.39, 54.04 and 69.09 mL/min • 1.73 m^2^, respectively), indicating that their GFR estimation was worse than eGFR7 (Figure 3-A, B, and C-F). Among eGFR1-6, eGFR3 and eGFR4 showed an apparent rightward shift in their error distribution curves (*P*_5_, *M* and *P*_95_ were all larger than eGFR7) (Figure 3-C and D), indicating overall underestimation of GFR by these two.

Similar results to those reported with eGFR7 were obtained with eGFR8 and all 6 previously reported equations (therefore, not shown, charted repeatedly). As shown in Figure 3-G, the deviation curves of eGFR7 and eGFR8 almost overlapped, indicating basically the same consistency in estimating the GFR.

Table 5 shows how many patients(percentage) were correctly classified for the different stages of CKD, according to GFR estimating equations based upon SCr and/or CysC. Overall a correct classification was achieved in 43.6% to 65.3% of the patients with the traditional eGFR equations and 73.3% to 74.3% with the new eGFR equations. The best percentage (74.3%) was achieved with the eGFR8, however, the eGFR7 gave a very close result 73.3%.

**Table 5:** Classification of CKD by SCr- and/or CysC-based on eGFR equations(n = 101)*.

	CKD1	CKD2	CKD3	CKD4	CKD5	Total	κ(95%CI)
rGFR	13	22	44	17	5	101	
eGFR1	7(53.8)^★^	14(63.6)	28(63.6)	5(29.4)	2(40.0)	56(55.4)	0.403(0.280–0.526)
eGFR2	7(53.8)	15(68.2)	19(43.2)	3(17.6)	2(40.0)	44(43.6)	0.297(0.175–0.419)
eGFR3	10(76.9)	17(77.3)	26(59.1)	7(41.2)	3(60.0)	63(62.4)	0.504(0.382–0.626)
eGFR4	11(84.6)	17(77.3)	29(65.9)	5(29.4)	2(40.0)	64(63.4)	0.514(0.394–0.634)
eGFR5	12(92.3)	14(63.6)	28(63.6)	9(52.9)	3(60.0)	66(65.3)	0.536(0.413–0.659)
eGFR6	11(84.6)	14(63.6)	27(61.4)	7(41.2)	2(40.0)	61(60.4)	0.472(0.347–0.597)
eGFR7	12(92.3)	18(81.8)	32(72.7)	9(52.9)	3(60.0)	74(73.3)	0.641(0.527–0.755)
eGFR8	12(92.3)	18(81.8)	33(75.0)	10(58.8)	2(40.0)	75(74.3)	0.652(0.538–0.766)

* Accoding to the recommendations by K/DOQI. ^★^Number of patients (%) correct classified. Reference method is plasma clearance of ^99m^Tc-DTPA. Kappa analysis was used to evaluate the agreement between rGFR stages and each eGFR CKD stages.

## Discussion

GFR is traditionally considered to be the best overall index of the kidney function. Therefore, its accurate detection is critical for early diagnosis, proper staging, effective treatment and monitoring of CKD. The International Association of Nuclear Medicine has recommended the two-serum method to measure the clearance rate of ^99m^Tc-DTPA as the reference method for GFR detection [35]. However, this method is difficult for clinical applications because of its complexity, high costs, high equipment requirement and radioactivity.

The PETIA-CysC is comparable with PENIA-CysC in accuracy, but PETIA-CysC can be detected using various types of automatic biochemical analyzers, so its detection speed is faster than PENIA-CysC and it can be easily and broadly applied in clinical laboratories. Therefore, many clinical laboratories have detected CysC using the PETIA-CysC method [36]–[40]. In addition, enzymatic detection of serum Cr shows less interference and low cross-contamination between samples [26]–[28]; therefore, it is also widely used by clinical laboratories.

In this study, we used the two-serum method ^99m^Tc-DTPA clearance rate as the “gold standard” and determined serum Cr with the enzyme-based method. We determined the serial CysC of 687 randomly selected Chinese CKD patients using the PETIA method. Two GFR estimating equations were established based on the top coefficient of the non-linear regression iterative calculation:
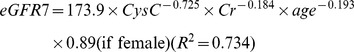


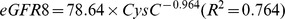



The R^2^ of eGFR8 was higher than that of eGFR7, indicating that using both Cr and age for GFR estimation was not as effective as using CysC alone. Therefore, CysC has the potential to be a good substitute for Cr during the assessment of renal function.

The two equations developed in this study had limits of agreement (57.0 mL/min • 1.73 m^2^ and 59.5 mL/min • 1.73 m^2^, respectively) that were within the pre-set values of <60 mL/min • 1.73 m^2^. They also showed only minor differences in their Mountain deviation distribution curves, with almost the same error (367 vs. 377 arbitrary units), precision (28.5 vs. 29.7 mL/min • 1.73 m^2^), accuracy (*P*>0.05), and applicability in GFR estimation. When comparing the newly developed equations and the 6 previously reported ones, only eGFR7 and eGFR8 did not show apparent proportional errors (b's 95% CI contains B = 1) and constant errors (a's 95% CI contains A = 0) from rGFR. All equations showed high linear correlations (r>0.75, *P* = 0.000) and acceptable Passing & Bablok regression linearity (Cusum test, *P*>0.05). Among these 6 previous equations, eGFR3 had only constant errors (a's 95% CI = −12.592 and −2.989, not containing 0) from rGFR, but all the other equations had both constant errors and proportional errors. These errors may have been caused by the differences in study subjects, GFR “gold standards”, GFR markers, and GFR determination methods, which may be verified by the following comparisons of the newly developed equations and the literature equations.

In the estimation of GFR consistency, eGFR7 and eGFR8 were both better than eGFR1 and eGFR2 (limits of agreement = 57.0 and 59.5 vs. 98.7 and 111.5 mL/min • 1.73 m^2^, respectively), and the limits of consistency for the latter two were both significantly higher than the pre-set professional cutoff value (<60 mL/min • 1.73 m^2^). eGFR7 and eGFR8 also had smaller GFR estimating errors than eGFR1 and eGFR2 (367 and 377 vs. 2045 and 3036 arbitrary units, respectively). eGFR1 and eGFR2 also had worse estimation accuracy (CR = 49.3 and 55.8 *vs.* 28.5 and 29.7 mL/min • 1.73 m^2^, respectively) and lower P_30_ and P_50_ than eGFR7 and eGFR8 (*χ* ^2^ test, *P*<0.05). The serum Cr can be easily affected by many factors, such as gender, age, muscle size, diet, medication and renal secretion and excretion (renal excretion becomes more apparent under pathological conditions), which may lead to the large errors and low accuracy of eGFR1 and eGFR2 in GFR estimation. In addition, the choice of Cr determination may also affect the applicability of eGFR1 and eGFR2. It was reported that eGFR1 was better than eGFR2 when the enzyme-based measurement was selected [41]. A similar conclusion was reached in this study.

The two equations for MDRD/CKD-EPI, eGFR3 and eGFR4, with the limits of agreement being higher than the pre-set values of <60 mL/min • 1.73 m^2^ at 61.8 and 72.3 mL/min • 1.73 m^2^, respectively, were also worse than eGFR7 and eGFR8 in the GFR estimation for Chinese CKD patients. The Mountain deviation distribution curves of eGFR3 and eGFR4 both deviated to the right from the “0” point (M = 6.90 and 6.63 mL/min • 1.73 m^2^, respectively,) with larger *P*_5_, *M* and *P*_95_ than those of eGFR7 and eGFR8, indicating significant underestimation of GFR for Chinese CKD patients. Therefore, the MDRD/CKD-EPI equations may only have limited application in Chinese populations.

The two equations from the Chinese cooperative group, eGFR5 and eGFR6, did target the Chinese populations. However, their limits of agreement (65.6 and 80.8 mL/min • 1.73 m^2^, respectively,)) both exceeded the pre-set values of <60 mL/min • 1.73 m^2^. Compared with eGFR7 and eGFR8, eGFR5 and eGFR6 showed broadening trends in the Mountain deviation distributions (*P*_5_–*P*_95_ interval distribution width = 54.04 and 69.09 vs. 48.94 and 49.94 mL/min • 1.73 m^2^, respectively,)), worse precision (CR = 32.8 and 40.4 vs. 28.5 and 29.7 mL/min • 1.73 m^2^, respectively,)), and worse accuracy (P_30_: eGFR6 *vs.* eGFR7, *χ* ^2^ test, *P*<0.05; P_50_: eGFR6 *vs.* eGFR7 and eGFR8, *χ* ^2^ test, *P*<0.05), all of which indicate inferior consistency for rGFR.

In addition to the factor of race, differences in the determination methods for GFR markers, i.e., the different methods of detecting Cr and CysC, may also contribute to the deviation of the results between the newly developed equations and the previously reported ones. In the past, the picric acid assay was used to detect serum Cr (eGFR1-eGFR6 equations). Currently, many laboratories in China prefer the enzyme-based method to detect serum Cr in order to avoid cross contamination from picric acid. Routine CysC tests also include the PETIA and PENIA methods. Because it can be performed in an automatic biochemical analyzer and the detection time can be as short as 5 minutes, PETIA has become the preferred method for CysC routine determinations. The MDRD/CKD-EPI and the Chinese collaboration group, however, used PENIA instead of PETIA to detect serum CysC (eGFR3-eGFR6 equations). In addition, the differences in CysC standards may also cause differences in detection results, which affect the applicability of eGFR equations. The first CysC certified reference material ERM-DA471/IFCC [42] was introduced in 2010, which allows CysC detection to be traced. As a result, studies on CysC-based GFR estimation equations may avoid matrix effects. Once serum CysC detection becomes standardized, its application in GFR evaluation will have greater advantages over Cr detection. In this study, we developed two eGFR equations based on the sole-indicator, CysC, in combination with serum creatinine levels and age, to evaluate renal functions precisely, accurately, simply and quickly with lower costs.

The difference of results between our new equation and traditional eGFR equations, largely may be due to different measured methods for the determination of SCr and CysC. That is, entered enzymatic-Cr results and/or PETIA-CysC results to traditional eGFR equations that were developed by Cr using the Jaffe's kinetic method and/or PENIA-CysC. It is this inadequate use that leads to the eGFR calculation resulting error. Of course, different choices of the "gold standard" in research programs, as well as technical proficiency of the same "gold standard" may also be the reasons of the error. In addition, the population differences in development group, such as the difference of the constituent ratio of the subjects age, sex, CKD stage, and complications, etc., may also be the reason that the results are not consistent between the traditional eGFR equations and this study equation. In this study, there are statistical differences between gender for weight, height, and body surface area. It will worth considering whether they will cause the differences of the traditional eGFR results.

It is worth mentioning that although the meta-analysis demonstrated that CysC is superior to SCr in the determination of the GFR injuries [43], [44]. However, most studies of CysC have focused on fields where the problems of SCr are most apparent, including specific population groups with malnutrition, extensive reduced body surface area, extremely low body mass, or a few comorbidities that have a large influence on the generation of creatinine. Nevertheless, Serum cystatin C may be influenced by factors other than renal function alone, including serum C-reactive protein [45], smoking [46], the subjects with very low GFR[47], thyroid function [48], [49], immunosuppressive therapy [50], and occupational exposure to toxic agents such as lead, cadmium, and arsenic [51], etc. Thus, clinicians must be cautious when interpreting cystatin C levels alone if the subjects encounter these factors.In summary, different fitting eGFR equations with different estimation values can be developed based on different study subjects, different GFR “gold standards”, and different detection methods. The two newly established equations in this study showed effective but significantly different results from previously reported equations, most likely a result of the differences in detection methods and “gold standards”. Currently, enzyme-based Cr detection and CysC PETIA detection are widely used in clinical laboratories. Our study demonstrated that the simple cystatin C formula could achieve a much better diagnostic performance than SCr formula containing more variables.Therefore, careful and complete consideration is required to choose the right eGFR equation for clinical applications to avoid misdiagnosis and errors in treatment.

## Supporting Information

Appendix S1 Representative patient's signed permit for Glomerular Filtration Rate(GFR) Measurement Using Radiochemical 99mTc-DTPA in Chinese Version.
(PDF)

Appendix S2 Representative patient's signed permit for publication in PLoS ONE in English Version.
(PDF)
