# Supplementary material for: Multicenter Study of Creatinine- and/or Cystatin C-Based Equations for Estimation of Glomerular Filtration Rates in Chinese Patients with Chronic Kidney Disease
Source: PLoS One. 2013 Mar 19;8(3):e57240. doi: 10.1371/journal.pone.0057240 (PMC3602457; doi:10.1371/journal.pone.0057240)
Supplement: Appendix S1 — Representative patient's signed permit for Glomerular Filtration Rate(GFR) Measurement Using Radiochemical 99mTc-DTPA in Chinese Version. (PDF) [file pone.0057240.s001.pdf]

放射性物质 ^{99m}Tc-DTPA 测定肾小球滤过率（GFR）

知情同意书

姓名： **王启明**　　性别： **男**　　年龄： **51**　　病历号：**6010597837**

一、疾病介绍和治疗建议：

医生已告诉我被诊断患有慢性肾病，为进一步确定病情，并对疾病严重程度进行分期，以达最佳治疗效果，需要进行放射性物质 ^{99m}Tc-DTPA 肾小球滤过率（GFR）测定。

二、诊疗潜在的风险和对策

医生已告诉我该检查技术已写入中华医学会《临床诊疗指南》（核医学分册）(人民卫生出版社，2006 年，北京)（P370-372）。

医生已告诉我该检查已获得医院伦理委员会批准。

医生告知我针对 ^{99m}Tc-DTPA 测定 GFR 的必要性及其可能存在的风险，有些不常见的风险可能没有在此列出，这根据不同的病人可能有所不同，医生告诉我可与我的医生讨论有关我该放射性检查的具体内容，如果我有特殊的问题可与我的医生讨论。

我理解实施本医疗方案可能发生的风险和医生的对策。

我理解全面的检查，有利于综合评估我的病情，有利于制定更恰当的治疗方案。

我理解不全面的检查评估将对我所患者的治疗方案的合理制定产生一定影响。我理解现有的医疗水平条件的限制，有可能出现不可预见的某些风险。

我理解对于辅助该 GFR 检查的其它实验室检查，如肾功能检查（尿素、肌酐、胱抑素 C、血糖），如需要进行上述及其它辅助检查时，医务人员将及时与我沟通，我将积极配合。

我理解根据我个人的病情，我可能出现未包括在上述所交待并发症意外的风险。

一旦发生上述风险和意外，医生会采取积极应对措施。

三、患者知情选择

我的医生已经告知我将要进行 GFR 检查及可能发生的风险，可能存在的其它检查方法，并且解答了患者关于此次治疗的相关问题。

我同意在诊疗过程中医生根据我的病情进行放射性物质 ^{99m}Tc-DTPA 测定 GFR 检查。

我并未得到治疗百分之百成功的许诺。

患者签名 [illegible] 签名日期 2011 年 2 月 7 日

如果患者无法签署知情同意书，请其授权的亲属在此签名：

患者授权亲属签名________与患者关系______签名日期 ____年____月____日

四、医生陈述

我已经告知患者将要进行 GFR 检查及可能发生风险，可能存在的其它检查方法，并且解答了患者关于此次治疗的相关问题。

医生签名 [illegible] 签名日期 2011 年 2 月 7 日

绵阳市中心医院

肾病科制
