# Supplementary material for: Multicenter Study of Creatinine- and/or Cystatin C-Based Equations for Estimation of Glomerular Filtration Rates in Chinese Patients with Chronic Kidney Disease
Source: PLoS One. 2013 Mar 19;8(3):e57240. doi: 10.1371/journal.pone.0057240 (PMC3602457; doi:10.1371/journal.pone.0057240)
Supplement: Appendix S2 — Representative patient's signed permit for publication in PLoS ONE in English Version. (PDF) [file pone.0057240.s002.pdf]

Appendix S2 Representative patient's signed permit for publication in PLoS ONE in English Version.

PLoS PUBLIC LIBRARY of SCIENCE

# Consent Form for Publication in a PLoS Journal

I, the undersigned, give my consent for my photograph and/or case history to be published in a Public Library of Science (PLoS) Journal. I have seen and read the material to be published. I have discussed this consent form with Jia-fu Feng, who is an author of this paper, and I understand the following:

All PLoS journals are freely available on the web[1]. Hence, anyone anywhere in the world can read material published in them. Readers include not only doctors, but also journalists and other members of the public.

My name will not be published, and as far as possible all identifying features will be removed. However, it is not possible to ensure complete anonymity, and someone may be able to recognize me.

I understand that under the license which the PLoS uses (the Creative Commons Attribution License[2]) material published in PLoS journals can be redistributed freely and used for any legal purpose, including translation into other languages and commercial uses. I also understand that signing this consent form does not remove my rights to privacy.

Name Cai-gao Zheng

Date Oct. 29, 2012

Signed Zhengcaigao

Author Jia-fu Feng

Date Oct.29,2012

Signed Fengjiafu

[1]PLoS Journals: http://www.plos.org/journals/

[2]Creative Commons Attribution License: http://creativecommons.org/licenses/by/2.5/

Please complete this form, obtain the patient's signature, and file in case notes.

The manuscript reporting this patient's details should state that consent to publication was obtained from the patient.
